# A prospective, triple-blind, randomized controlled clinical trial evaluating the protective effect of P11-4 peptide on enamel demineralization during multibracket orthodontic treatment

**DOI:** 10.1186/s12903-025-07642-3

**Published:** 2026-01-09

**Authors:** Anahita Jablonski-Momeni, Teresa Temming, Jan-Niklas Berthold, Carolin Olbrisch, Caroline Diekmeier, Peter Bottenberg, Heike Korbmacher-Steiner

**Affiliations:** 1 Dental School, Department of Orthodontics, Philipps-Universität Marburg, Georg-Voigt Str. 3, Marburg, 35039 Germany; 2 Department of Oral Health Care, Université Libre de Bruxelles, Route de Lennik 808, Brussels, 1070 Belgium; 3 Vrije Universiteit Brussel, Laarbeeklaan 103, Brussels, 1090 Belgium

**Keywords:** Orthodontics, Enamel demineralization, P11-4 peptide, Fluoride, Multibracket appliances, QLF, Caries prevention

## Abstract

**Background:**

This prospective, triple-blind, randomized controlled clinical trial evaluated the additional caries-preventive effect of a self-assembling peptide (P11-4) gel with fluoride applied at home during multibracket (MB) orthodontic treatment in adolescents.

**Participants and methods:**

Twenty-five patients aged 11–17 years (mean age 14.5 years) were randomized to a test group (P11-4 gel including 900 ppm sodium monofluorophosphate) or a placebo group (900 ppm sodium monofluorophosphate gel without P11-4 gel). Participants applied the assigned gel twice weekly for 18 months. Caries development was assessed using the International Caries Detection and Assessment System (ICDAS) and quantitative light-induced fluorescence (QLF) at baseline (t0), and after 6 (t1), 12 (t2), and 18 months (t3) after baseline. Statistical analyses were performed using non-parametric tests (α = 0.05).

**Results:**

At 18 months, 7.2% of surfaces in the test group and 14.5% in the placebo group showed initial lesions (ICDAS 1 or 2; *p* < 0.0001). All QLF parameters showed significantly lower demineralization in the test group throughout the study (*p* < 0.0001). In the test group, QLF parameters at t3 were: ΔF = -0.91%, ΔFmax = -1.17% and ΔQ = -12.06%px. Corresponding values in the placebo group were: ΔF = -2.07%, ΔFmax = -2.75% and ΔQ = -36.31%px. Lesion volume (ΔQ) decreased over time in the test group, indicating remineralization. No adverse effects were observed.

**Conclusion:**

Regular home use of P11-4 gel with fluoride reduced the development of enamel demineralization in adolescents with fixed appliances. Despite the small sample size and limited patient-level power, the findings indicate that P11-4 with fluoride can serve as a useful adjunct to established home-based preventive measures during orthodontic treatment.

**Clinical relevance:**

A biomimetic peptide gel with fluoride used at home can prevent development of initial caries lesions during orthodontic treatment and may benefit patients with limited oral hygiene or high caries risk.

The study was conducted in accordance with the World Medical Association Declaration of Helsinki. The study protocol was reviewed and approved by the Ethics Committee of the Medical Faculty of the Philipps-University of Marburg, Germany (approval number 197/21, date of approval: 20 January 2022).

The study protocol was registered in the German Clinical Trials Register: Trial registration number: DRKS00028048, date of registry: 07 February 2022). The study was conducted following the Good Clinical Practice standards and the General Data Protection Regulation (GDPR).

## Background

Fixed multibracket (MB) appliances remain a fundamental tool in orthodontic treatment, enabling the precise correction of malocclusions through controlled tooth movement. Nonetheless, the use of MB systems has been associated with an increased risk of enamel demineralization [1, 2], particularly in the form of white spot lesions, which are now more appropriately referred to as initial caries lesions [3]. The development of these lesions is due to the formation of plaque-retentive niches around brackets and arch wires. These lesions can develop within a few weeks of appliance placement [4–7] and represent a significant clinical and aesthetic problem. The pooled estimates of incidence and prevalence have been reported as 45.8% and 68.4%, respectively [8]. Conventional preventive measures during orthodontic treatment, including intensive oral hygiene instructions, fluoride applications, and the use of antimicrobial agents, have shown varying degrees of success. Nevertheless, these approaches frequently depend on patient´s adherence to the treatment plan and may not fully address the underlying imbalance between demineralization and remineralization at the enamel surface. Consequently, there is an increased interest in the use of adjunctive agents that are capable of promoting enamel repair at an early stage.

Different caries preventive strategies can contribute maintaining or restore a balanced microbiome. These include sugar reduction, daily tooth brushing and use of fluorides [9]. The caries-preventive effect of fluorides is known to be achieved through the facilitation of remineralization, the inhibition of demineralization, and the interference with bacterial metabolism and plaque formation [10–12]. The effectiveness of various fluoride treatments in preventing the initial stages of caries lesions during orthodontic treatment with fixed appliances has already been demonstrated to some extent [13, 14]. Nevertheless, regular application of fluorides is necessary. It was demonstrated that the one-time application of fluoride containing agents would not yield any additional preventive advantage over sufficient domestic dental hygiene with fluoride toothpaste in patients with a low to moderate caries risk [15]. Data from a systematic review suggested that the professional hygiene and prophylaxis employed in the prevention of plaque accumulation in orthodontic patients was generally efficacious [16].

In recent years, a range of other potential measures for the prevention of initial lesions has been the focus of investigation. These include casein phosphopeptide-amorphous calcium phosphate fluoride (CCP-ACPF) [17], probiotics [18] laser irradiation [19], and the use of coating agents like sealants or bonding materials around brackets [20, 21].

A novel approach in the field of preventive dentistry is represented by the self-assembling peptide P11-4, a material designed to mimic the natural process of enamel remineralization. The underlying mechanism has previously been demonstrated [22–25]. In brief, subsequent to the application of P11-4, a pH-triggered self-assembly process occurs, resulting in the formation of a three-dimensional scaffold within the subsurface lesion. This serves as a template for the nucleation and growth of hydroxyapatite crystals. This mechanism has been demonstrated to stabilize early carious lesions and to reverse the process of demineralization without the necessity for invasive interventions [26]. Since the introduction of P11-4, numerous studies have been performed and the outcomes seem supportive of an effect on arresting and shrinking initial caries lesions as measured by means of various criteria such as visual-tactile assessment, fluorescence measurements, digital photography and radiography [26]. In orthodontics, the P11-4 was shown to be effective in the treatment of initial carious lesions [27–29].

A variety of products are available that contain the P11-4 (vVvardis AG, Zug, Switzerland). Curodont™ Repair and Curodont™ Repair Fluoride Plus have been formulated as liquids for utilization in dental offices; meanwhile, Curodont™ Protect has been formulated as a gel which can be applied both professionally and at home. In a clinical setting, it can be used after dental cleaning or bleaching, and during orthodontic follow-up appointments. For at-home use, patients can apply it up to twice a week after brushing teeth, allowing it to remain on the teeth without immediate rinsing to maximize contact time.

While studies have demonstrated the ability of Curodont™ Repair to promote remineralization in initial carious lesions [26], there is limited evidence regarding its effectiveness of Curodont™ Protect as a home-based preventive measure during the high-risk period of fixed orthodontic treatment. Therefore, the aim of the present prospective, randomized, blinded clinical study was to investigate the caries-protective effect of Curodont™ Protect with fluoride (from here referred to as P11-4 gel) when used as part of a home prevention regimen in adolescents undergoing MB treatment. By comparing the development and progression of demineralization between a test group using P11-4 gel with fluoride and a control group using a placebo agent without P11-4 but with fluoride, this study aimed to provide robust clinical data on the efficacy of this biomimetic approach.

## Participants and methods

### Trial design

The prospective, blinded, randomized controlled clinical trial was conducted in accordance with the World Medical Association Declaration of Helsinki. The study protocol was reviewed and approved by the Ethics Committee of the Medical Faculty of the Philipps-University of Marburg, Germany (approval number 197/21, date of approval: 20 January 2022).

The study protocol was registered in the German Clinical Trials Register (DRKS00028048, date of registry: 07 February 2022) and was conducted following the Good Clinical Practice standards and the General Data Protection Regulation (GDPR).

Written informed consent to participate was obtained from all participants and from the parents or legal guardians of those under the age of 18.

**Fig. 1:**
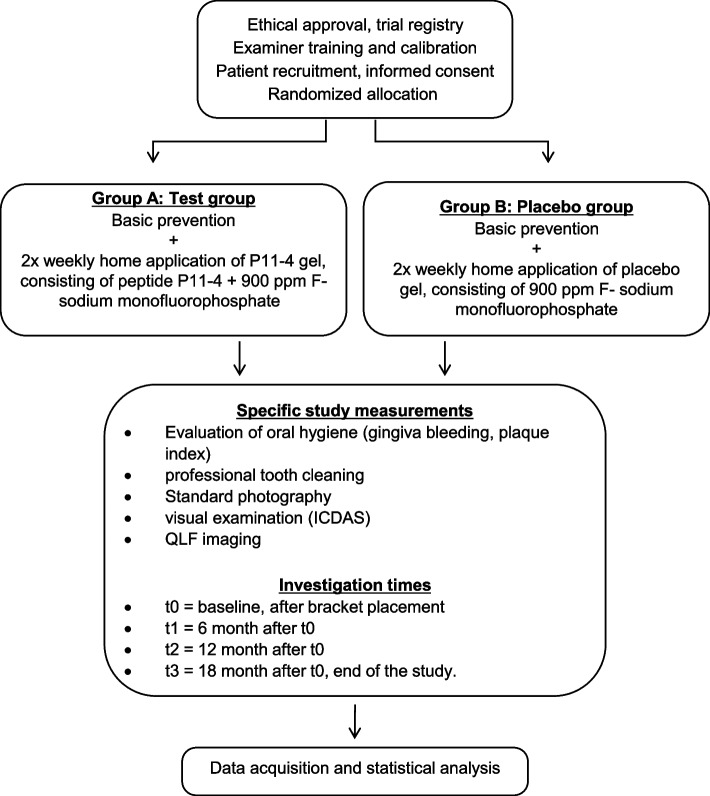
Flow diagram of the study design

A flow diagram of the study design is shown in Fig. 1.

### Sample size calculation

Sample size estimation was performed prior to the start of the study (PASS 2020, v20.0.5) according to a model for two group comparison. Considering an intraclass correlation coefficient (ICC) of 0.05, α = 0.05 and a power of 90%, 8 patients were calculated for each group. The cluster effect was taken into consideration, as a result of the fact that teeth belonging to the same patient are not independent of each other. To adjust for dropouts, the number of patients was increased by 20%, so that ten patients were planned for inclusion in each group.

### Participants, eligibility criteria, and settings

The clinical study enrolled patients who were scheduled to receive treatment for tooth position and/or bite abnormalities with a multibracket appliance (MB). Patients were checked for inclusion and exclusion criteria and were recruited between June 2022 and October 2023 for participation in the study. All patients were recruited from the Department of Orthodontics, Dental School, Philipps-University Marburg.

#### Inclusion criteria were:

- Age 11–18 years
- Patients with a scheduled multibracket appliance
- Written, signed informed consent from patient and legal guardian before the start of the study
- Individuals who are willing and able to attend all scheduled appointments
- Patients are willing and able to understand all study-related procedures and follow home-care instructions.

#### Exclusion criteria were:

- Individuals with removable dentures
- Individuals with recent dental trauma or dental surgery
- Patients with current periodontal disease
- patients with bronchial asthma
- Signs of tooth erosion (excessive drinking of acidic drinks, reflux)
- Medical conditions or concomitant medication that affect salivation or cause dry mouth
- Last antibiotic intake < 2 months
- Use of products that can cause tooth discoloration (e.g., chlorhexidine digluconate)
- Patients with known allergies/hypersensitivity to the used materials
- Pregnant or breastfeeding women.

All patients lived in an area where the concentration of fluoride in the tap water was up to 0.25 mg/L. This level remained constant for many years.

### Randomization

Patients were randomly allocated to either the test group (A) or the placebo group (B). A randomization list was generated using MedCalc Statistical Software, v20.019 (Ostend, Belgium; www.medcalc.org), and the computer-generated list was stored in the secretary’s office of the Department of Orthodontics. Opaque, consecutively numbered, sealed envelopes were prepared, each containing the group assignment (A or B). After enrollment, the next envelope in sequence was opened by the secretary, who also provided the allocated study materials. This process implemented a concealed, sequential randomization procedure, ensuring allocation concealment and preventing foreknowledge of upcoming assignments. In addition, a decoding key list was prepared and securely stored in the secretary’s office, accessible only after completion of data collection and finalization of the dataset to preserve blinding.

**Fig. 2:**
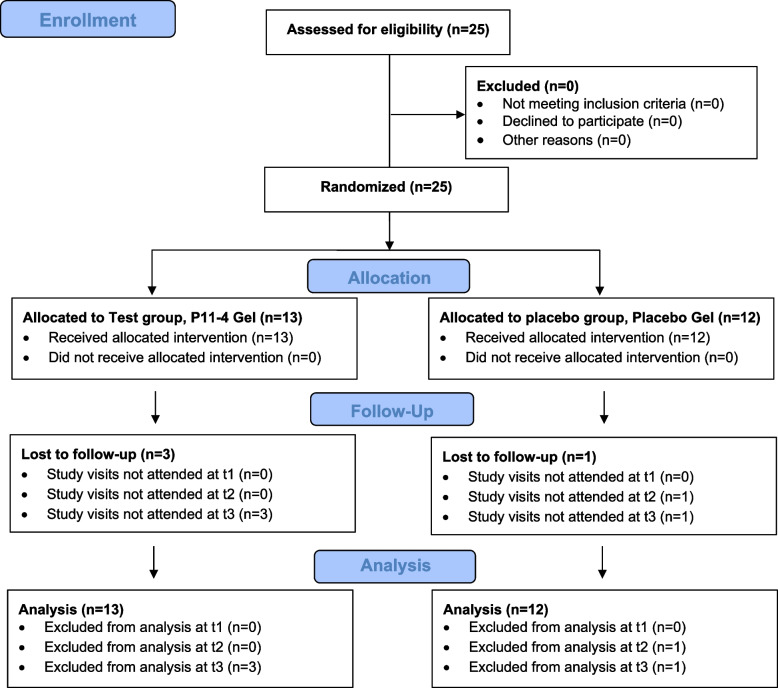
Consolidated Standards of Reporting Trials (CONSORT) flow diagram of patient selection and allocation

A Consolidated Standards of Reporting Trials (CONSORT) flow diagram illustrating patient selection and allocation is presented in Fig. 2.

### Blinding

This trial was conducted in a triple-blind design. Neither investigators, patients, nor their legal guardians were informed of group allocation. Fluorescence images were analyzed by an examiner not involved in treatment or imaging, and statistical analyses were performed without knowledge of group assignment. To maintain blinding, all study materials were transferred via the secretary’s office, and blinding was maintained until all analyses were completed.

### Intervention

Patients were randomized into two groups for home care prevention measures:

- Group A (test group): 2 × weekly home application of the test gel (Curodont Protect), consisting of peptide P11-4 + 900 ppm sodium monofluorophosphate.
- Group B (placebo group): 2 × weekly home application of the placebo gel without P11-4 (ingredients: sorbitol, aqua, hydrated silica, propylene glycol, cellulose gum, 900 ppm sodium monofluorophosphate, flavoring, sodium saccharin, sodium methylparaben, limonene, CI 42090).

In addition to the study specific oral home care, all patients received every six months the basic prophylaxis established in our department for patients undergoing orthodontic treatment with a multibracket appliance (MB). This program consisted of professional tooth cleaning and the application of a fluoride varnish (elmex fluid, CP GABA GmbH, Hamburg, consisting of 10.000 ppm F^−^: Amine fluoride, Dectaflur, Olaflur).

For the regular dental care at home, the participants were provided with a prophylaxis package comprising a manual toothbrush specifically for cleaning braces, a soft toothbrush, interdental brushes in assorted sizes and fluoride toothpaste with 1450 ppm sodium fluoride (all materials: DentAid GmbH, Mannheim, Germany). Patients were instructed in tooth brushing techniques and practiced their use. Patients were advised to brush twice a day for 2–3 min using the materials provided. The use of other dental care products containing fluoride or other active ingredients should be avoided. Each participant received a report form to document the application of their assigned gel. New gel tubes and prophylaxis packages were handed over on a six-month basis to ensure consistent utilization.

### Study specific investigation times

Prior to the insertion of the brackets, a thorough examination of the teeth was performed. However, the time at bracket insertion was determined as the baseline, ensuring that all participants were subject to equivalent conditions and enabling a comparison of the measurements from baseline to the end of the study. Study specific measurements were conducted at the following investigation times:

- *t0* = *baseline, after bracket placement*
- *t1* = *6 months after t0*
- *t2* = *12 months after t0*
- *t3* = *18 months after t0 (end of the study)*

The regular orthodontic control and treatment intervals were not affected by the study periods. After the end of the study (18 months after bracket placement), orthodontic treatment was continued as needed.

### Termination criteria

Termination at the patient's or their legal guardian's request was permitted. Also, cancellation by the practitioner was permitted in the event of the patient failing to cooperate with treatment, as indicated by two or more missed appointments per quarter.

### Fluorescence measurements (primary outcome)

At each investigation time, standardized images were produced using quantitative light-induced fluorescence (QLF) as a clinical reference standard (Qraycam Pro, Inspektor Research Systems B.V.). QLF images were evaluated by the proprietary software (C4 Research software, v.1.08). Fluorescence behavior (ΔF, ΔFmax and ΔQ) of the surfaces were analyzed in the “whitespot analysis mode” by an investigator who was not involved in the clinical examinations at a later time.

According to the manufacturer´s instruction for use, ΔF (%) corresponds to the average fluorescence loss in a surface and is related to the loss of mineral content and to lesion depth. A higher negative number indicates a deeper lesion. ΔFmax (%) displays the highest value of ΔF measured within the region of interest and is an indication of the maximum lesion depth. ΔQ (%px) corresponds to lesion volume and is the integral of ΔF over the area of the investigation site in pixels. Higher values of ΔQ indicate a larger lesion.

### Examiner background and training for visual assessment ICDAS (secondary outcome)

Two orthodontists participated in the clinical examinations. Prior to the study, the examiners underwent training in the use of the visual classification system ICDAS (International Caries Detection and Assessment System) [30–32]. The training was performed by an experienced clinician with a cariology background and qualification in the use of ICDAS and various other caries detection and assessment methods. To ensure the quality of the data assessment, intra- and inter-examiner agreements (Kappa values) were analyzed prior to the clinical examinations. Clinical images of patients who were not involved in the study were used for training and calibration purposes. After theoretical training and after completing an eLearning session video (https://www.iccms-web.com/), images of 60 teeth with and without orthodontic brackets were independently examined twice within one week using the ICDAS criteria for sound surfaces and detection of initial enamel caries lesions [32]:

- ICDAS code 0: sound tooth surfaces, no evidence of visible caries when viewed clean and after 5 s of air drying.
- ICDAS code 1 and 2: first or distinct visual changes in enamel seen as a carious opacity or visible discoloration (white spot lesion and/or brown carious discoloration) not consistent with clinical appearance of sound enamel, with no evidence of surface breakdown or underlying dentine shadowing.

Emphasis was given on the differentiation between caries lesions and other dental defects including erosion, fluorosis, MIH, or other developmental defects.

The kappa-values are summarized in Table 1, all corresponding to an almost perfect agreement [33].

**Table 1:** Kappa values for ICDAS examiner agreement prior to the clinical study (95% confidence interval in parenthesis)

	**Examiner 1**	**Examiner 2**
Reference examiner	0.83 (0.70 to 0.97)^a^	0.83 (0.68 to 0.96)^a^
Examiner 1	0.90 (0.78 to 1.00)^b^	0.93 (0.83 to 1.00)^a^
Examiner 2		0.82 (0.66 to 0.97)^b^

^a^inter-examiner agreement

^b^intra-examiner agreement

#### Assessment of oral hygiene parameters

Standardized oral hygiene parameters [30] were assessed at the beginning of each visit. First, sulcus bleeding index (SBI) was determined using a WHO probe. Then a plaque disclosing solution (Mira-2-Ton, Hager & Werken GmbH & Co. KG, Germany) was applied on the dried teeth and the approximal plaque index (API) was recorded. Teeth were cleaned afterwards using a rotating brush and fluoride free prophylaxis paste (Zircate® Prophy Paste, Dentsply De Trey GmbH, Constance, Germany) and the buccal surface of all accessible permanent teeth were examined.

For all methods, four sites around each bracket were examined: incisal, cervical, mesial, and distal. The second permanent molars and, when present, third molars were not included in the assessments.

### Statistical analysis

Statistical analysis was performed using MedCalc Statistical Software, v23.1.3. Data were tested for normal distribution using the Shapiro–Wilk’s test (*p* < 0.05) and non-parametric tests were used further. The correlation between the visual and fluorescent measurements was calculated by Spearman's correlation coefficient (rs). Group comparison for independent samples was performed by means of Mann–Whitney U test. Multiple comparisons between the different investigation times within each treatment group were performed using the Friedman test for dependent data. Post-hoc analyses followed the Conover-Iman Test of multiple comparisons using rank sums as implemented in MedCalc. Cohen´s d was calculated to determine the effect size for the assessed parameters. The significance level was set at α = 0.05.

#### Handling of missing data

Analyses were performed according to the intention-to-treat principle. Missing values at the tooth level were addressed by complete case analysis, i.e., only sites with available data at a given time point were included. The number of missing values per outcome and time point is reported in the result section.

## Results

The clinical study was conducted between June 2022 and October 2024. 25 adolescences (13 female, 12 male) were enrolled in the study. At the beginning of the study, the mean age of participants was 14.5 years (range 11.2–16.7 years), with no significant difference in age between participants in the two groups (*p* = 0.33).

13 participants (6 female, 7 male) were assigned to the test group (A) and 12 participants (7 female, 5 male) to the placebo group (B). Follow-up data at t3 (end of the study) were not available for 3 patients in group A and 1 patient in group B. Data from these patients were analyzed for all other examination times. There was no breach of protocol by any participant recorded. The baseline characteristics of the participants at study entry are presented in Table 2.

**Table 2:** Baseline characteristics of participants at study entry (SD = standard deviation)

Characteristic	Test group (*n* = 13)	Placebo group (*n* = 12)	*p*-value
Mean Age in years (SD)	14.8 (1.3)	14.25 (1.4)	0.33
Sex, n female/male	6/7	7/5	0.56
Mean number of teeth (SD)	20 (2.4)	20 (2.9)	1.00
Mean API in %(SD)	29.7 (0.11)	30.1 (0.11)	0.94
Mean SBI in % (SD)	3.5 (0.04)	2.9 (0.06)	0.78

### Adverse events

No adverse effects were reported by any of the patients during the trial.

#### Number of teeth and surfaces

In total, 1025 surfaces (n teeth = 257) in group A and 946 surfaces (n teeth = 237) in group B were examined at t0. Detailed data about the number of investigation sites at different investigation times are displayed in Table 3.

**Table 3:** Number of examined teeth and tooth surfaces at different investigation times (SD: standard deviation)

	**Group A = Test group**		**Group B = Placebo group**	
	N total	Mean number (SD)	N total	Mean number (SD)
Participant t0	13		12	
Teeth t0	257	20 (2.4)	237	20 (2.9)
Surfaces t0	1025	146 (9.6)	946	79 (11.7)
Participant t1	13		12	
Teeth t1	253	19 (2.5)	226	19 (2.9)
Surfaces t1	1010	144 (9.9)	903	75 (11.7)
Participant t2	13		11	
Teeth t2	252	19 (2.8)	209	19 (2.1)
Surfaces t2	1007	144 (11.3)	834	76 (8.5)
Participant t3	10		11	
Teeth t3	204	20 (2.1)	218	20 (1.7)
Surfaces t3	816	148 (8.6)	870	79 (6.7)

### Results of oral hygiene assessments

**Fig. 3:**
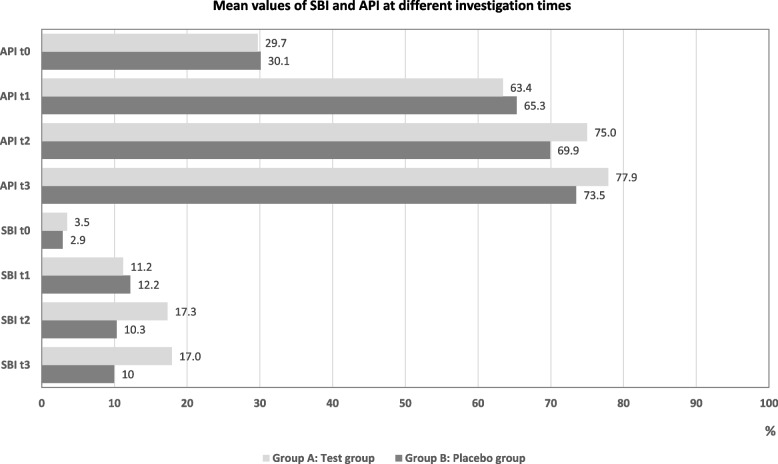
Mean values of SBI and API at different investigation times

Mean values of oral hygiene scores at different investigation times are presented in Fig. 3. At baseline, oral hygiene parameters were comparable between participants in both groups (Table 2). During the study, oral hygiene indices showed a significant increase from baseline in both groups (*p* < 0.01). However, no statistically significant differences were observed between the two groups by the end of the study: *p* = 0.11 for SBI and *p* = 0.63 for API.

### Results of quantitative light-induced fluorescence (QLF)

At baseline, all fluorescence values were zero, corresponding to a sound surface. During the treatment period, the values changed towards demineralization (below 0). At t1, 1,4% of the tooth surfaces in group A had maximum ΔF values below −12%, corresponding to first clinical changes in the enamel [34]. This proportion was 3.2% in group B (*p* = 0.007). At t2, 2.4% of the surfaces in group A and 4.6% in group B showed signs of demineralization (*p* = 0.010). At the end of the study (t3), 2.3% of group A and 4.8% of group B showed fluorescence loss (*p* = 0.006).

**Fig. 4:**
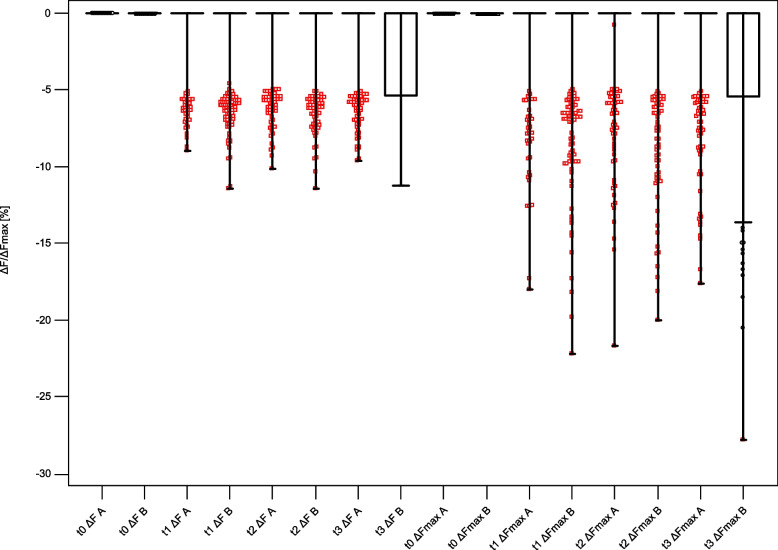
Boxplots of ΔF and ΔFmax values for both groups at all investigation times. **A** = Test group, **B** = Placebo group

**Fig. 5:**
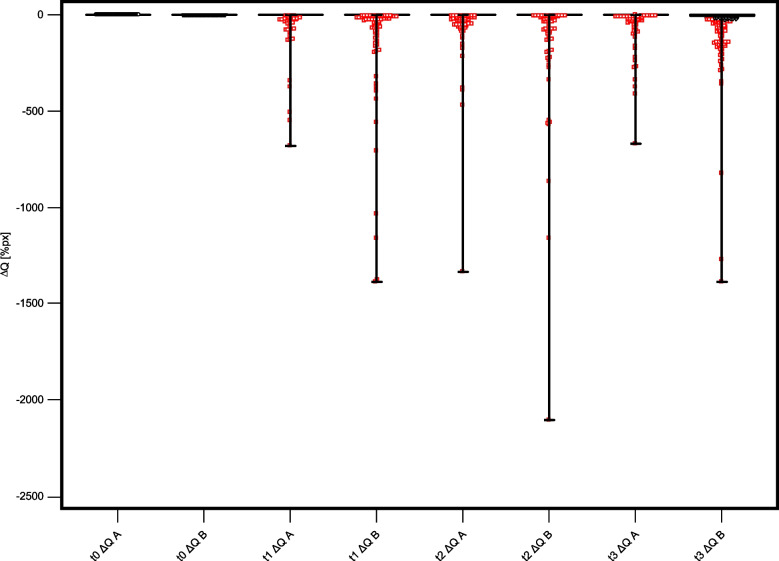
Boxplots of ΔQ values for both groups at all investigation times. **A** = Test group, **B** = Placebo group

The results of all QLF measurements from t1 to t3 are summarized in Table 4. The corresponding box-plots for all QLF measurements are presented in Figs. 4 and 5.

**Table 4:** Results of the QLF measurements in both groups at different investigation times (CI: confidence interval)

	**Group**	**Minimum**	**Maximum**	**Mean**	**95% CI**	**Median**	**Group comparison**
t1 ΔF [%]	A = Test	−17.3	0	−0.77^a^	−0.90 to −0.63	0	*p* < 0.0001
	B = Placebo	−79.0	0	−1.52^b^	−1.77 to −1.27	0	
t1 ΔFmax [%]	A = Test	−34.2	0	−1.01^d^	−1.21 to −0.81	0	*p* < 0.0001
	B = Placebo	−54.0	0	−1.97^e^	−2.26 to −1.68	0	
t1 ΔQ [%px]	A = Test	−3090.0	0	−16.00	−23.88 to −8.12	0	*P* < 0.0001
	B = Placebo	−1786.0	0	−27.51^g^	−35.73 to −19.30	0	
t2 ΔF [%]	A = Test	−12.1	0	−0.97	−1.12 to −0.82	0	*P* = 0.0001
	B = Placebo	−12.9	0	−1.47^c^	−1.66 to −1.28	0	
t2 ΔFmax [%]	A = Test	−57.0	0	−1.32	−1.56 to −1.08	0	*p* = 0.0001
	B = Placebo	−66.0	0	−2.06^f^	−2.39 to −1.74	0	
t2 ΔQ [%px]	A = Test	−1335.0	0	−15.21	−20.44 to −9.98	0	*p* < 0.0001
	B = Placebo	−2622.0	0	−35.11^h^	−46.21 to −24.00	0	
t3 ΔF [%]	A = Test	−11.1	0	−0.91^a^	−1.07 to −0.75	0	*p* < 0.0001
	B = Placebo	−11.5	0	−2.07^b,c^	−2.28 to −1.86	0	
t3 ΔFmax [%]	A = Test	−20.0	0	−1.17^d^	−1.39 to −0.96	0	*p* < 0.0001
	B = Placebo	−27.8	0	−2.75^e,f^	−3.06 to −2.44	0	
t3 ΔQ [%px]	A = Test	−846.0	0	−12.06	−16.04 to −8.09	0	*p* < 0.0001
	B = Placebo	−1875.0	0	−36.31^g,h^	−45.37 to −27.26	0	

^*^Different superscript letters indicate significant differences between the variables: a, b, c, d, e, f, g, h: *p* < 0.00001

Group comparison revealed significant differences (*p* < 0.0001) between the fluorescence behavior between the test and placebo group already after 6 months of study start (t1). The mean values of each fluorescence variable were higher in the test group than in the placebo group at each investigation time. This shows that demineralization increased significantly in the placebo group during the treatment period.

Multiple comparisons between different investigation times showed significantly lower fluorescence values at t3 compared to t1 in both groups (*p* < 0.00001). At t3, lesion volume in group A (ΔQ −12.06) showed a shift toward remineralization compared to t1 (ΔQ −16.0).

Cohen´s d (effect sizes) at t3 were: ΔF = −0.42 (95% CI −0.50;−0.39), ΔFmax = −0.40 (95% CI −0.49;−0.36), and ΔQ = −0.23 (95% CI −0.30;−0.20), respectively.

A post-hoc calculation using the observed effect size (Cohen’s d), α = 0.05 and the actual group sample sizes was performed in G*Power [35]. For ΔF, an estimated post-hoc power of 15% was calculated for the number of participants and 99% for the number of included teeth.

**Fig. 6:**
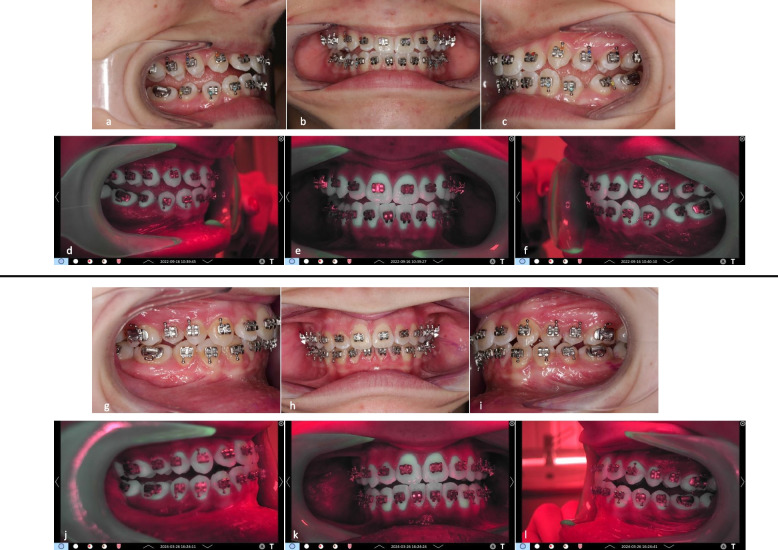
Representative clinical images of patient #06, group A: **a**-**c** lateral right, frontal, and lateral left images of the arches at t0; **d**-**f** corresponding QLF-images. At baseline, fluorescence values ΔF, ΔFmax and ΔQ values were zero (0). **g**-**i** lateral right, frontal, and lateral left images of the arches at t3; **j**-**l**: corresponding QLF-images. At t3, mean fluorescence values for this patient for all teeth were: ΔF = −0.79%, ΔFmax = −1.04%, ΔQ = −6.78%px

**Fig. 7:**
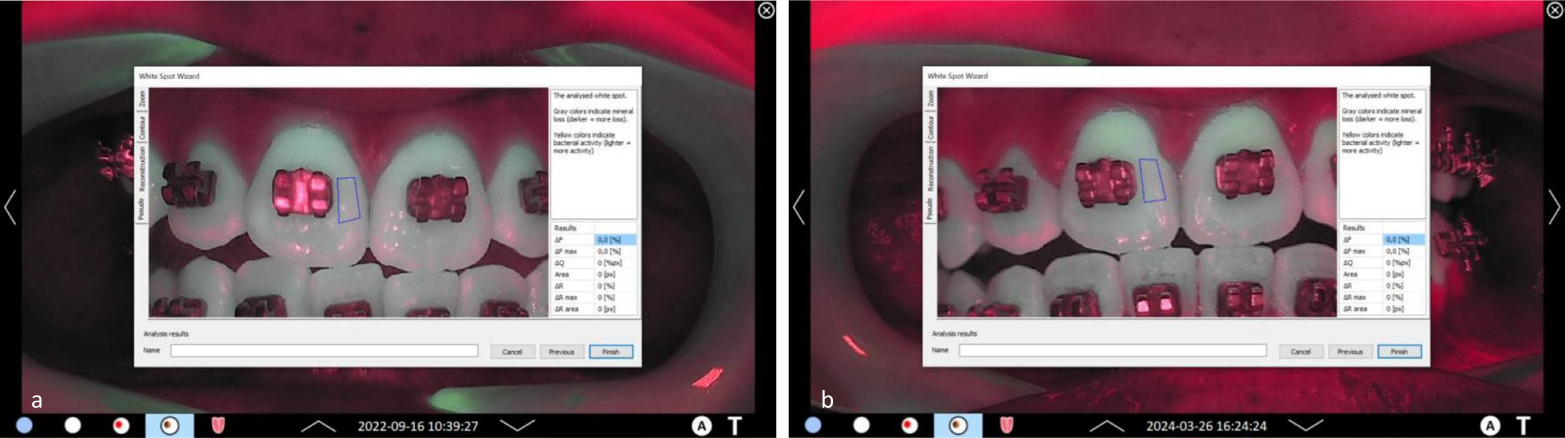
Representative QLF images of patient #06, group A in the “whitespot analysis mode”. The square indicates the surface where the QLF measurements were performed. **a** tooth 11 mesial site. At baseline, fluorescence values ΔF, ΔFmax and ΔQ values were zero (0). **b** same tooth and site at t3. As displayed, fluorescence values for the specific tooth site were: ΔF = 0%, ΔFmax = 0%, ΔQ = 0%px

**Fig. 8:**
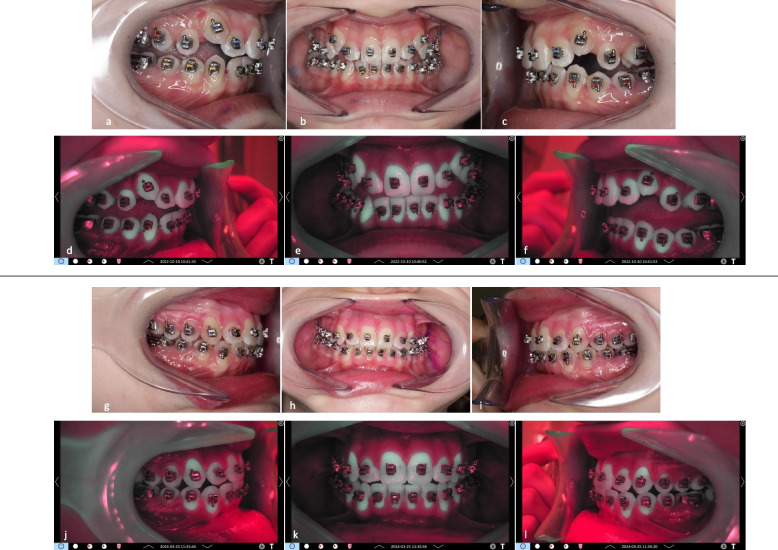
Representative clinical images of patient #08, group B: **a**-**c** lateral right, frontal, and lateral left images of the arches at t0; **d**-**f**: corresponding QLF-images. At baseline, fluorescence values ΔF, ΔFmax and ΔQ values were zero (0). **g**-**i** lateral right, frontal, and lateral left images of the arches at t3; **j**-**l**: corresponding QLF-images. At t3, mean fluorescence values for this patient for all teeth were: ΔF = −2.5%, ΔFmax = −3.31%, ΔQ = −43.99%px

**Fig. 9:**
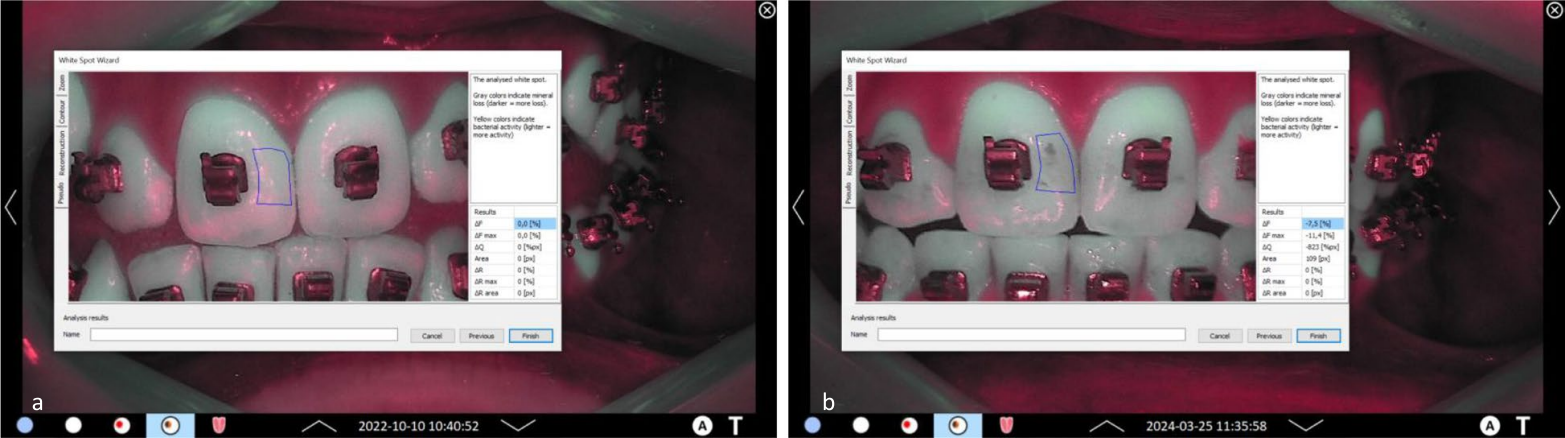
Representative clinical images of patient #08, group B. in the “whitespot analysis mode”. The square indicates the surface where the QLF measurements were performed. **a** tooth 11 mesial site. At baseline, fluorescence values ΔF, ΔFmax and ΔQ values were zero (0). **b** same tooth and site at t3. Fluorescence values for the specific site were: As displayed, fluorescence values for the specific tooth site were: ΔF = −7.5%, ΔFmax = −11.4%, ΔQ = −823%px

Figures 6, 7, 8 and 9 present clinical and fluorescence images from one patient in each group at t0 and t3, together with the corresponding QLF parameters for a representative tooth site.

Gender-specific analysis indicated differences in fluorescence values at t3. At t3, the gender distribution was in group A: 5 female, 5 male and in group B: 7 female and 4 male, respectively. In group A, no significant differences were observed for fluorescence values between male and female participants. In group B, female participants had significantly lower ΔF and ΔFmax values compared to the male (*p* < 0.0001), whereas ΔQ values did not differ significantly (*p* = 0.265) between male and female participants. Detailed data are given in Table 5.

**Table 5:** Results of the gender specific QLF measurements in both groups at t3 (CI: confidence interval)

**Group A**	**Gender**	**Minimum**	**Maximum**	**Mean**	**95% CI**	**Median**	**Group comparison**
t3 ΔF [%]	female	−11.1	0	−0.87	−1.09 to −0.64	0	*p* = 0.623
	male	−9.7	0	−0.95	−1.17 to −0.72	0	
t3 ΔFmax [%]	female	−20.0	0	−1.14	−1.45 to −0.82	0	*p* = 0.733
	male	−17.7	0	−1.21	−1.52 to −0.91	0	
t3 ΔQ [%px]	female	−846.0	0	−12.41	−18.63 to −6.19	0	*p* = 0.867
	male	−668.0	0	−11.73	−16.76 to −6.70	0	
**Group B**	**Gender**	**Minimum**	**Maximum**	**Mean**	**95% CI**	**Median**	**Group comparison**
t3 ΔF [%]	female	−11.2	0	−2.41	−2.69 to −2.14	0	*p* < 0.0001
	male	−11.5	0	−1.44	−1.74 to −1.13	0	
t3 ΔFmax [%]	female	−27.8	0	−3.27	−3.69 to −2.86	0	*p* < 0.0001
	male	−22.5	0	−1.80	−2.22 to −1.39	0	
t3 ΔQ [%px]	female	−1269.0	0	−40.43	−50.55 to −30.32	0	*p* = 0.265
	male	−1875.0	0	−28.84	−46.59 to −11.08	0	

Data analysis of all teeth showed that incisors were the tooth type most affected by demineralization at t3 (mean ΔF −2.01 ± 3.12), followed by canines (mean ΔF −1.58 ± 2.89), molars (mean ΔF −1.35 ± 2.78) and premolars (mean ΔF −0.91 ± 2.32). Surface based analyses in all patients revealed the mesial surface as the most frequent surface to develop demineralization at t3 (mean ΔF −1.97 ± 2.64), followed by the cervical (mean ΔF −1.38 ± 2.81), incisal (mean ΔF −1.32 ± 2.69) and distal surfaces (mean ΔF −1.31 ± 2.64).

Spearman correlation coefficients (rs) for the visual and fluorescent measurements were between −0.024 and −0.197. All data are summarized in Table 6.

**Table 6:** Spearman correlation coefficient (rs) and corresponding p-values for all measurements at different examination times

	**Group**	**t1 ICDAS**	**t1 ICDAS**	**t1 ICDAS**
t1 ΔF	A = Test	−0.054 (*p* = 0.102)		
	B = Placebo	−0.085 (*p* = 0.017)		
t1 ΔFmax	A = Test	−0.053 (*p* = 0.101)		
	B = Placebo	−0.085 (*p* = 0.017)		
t1 ΔQ	A = Test	−0.052 (*p* = 0.115)		
	B = Placebo	−0.086 (*p* = 0.015)		
t2 ΔF	A = Test		−0.197 (*p* < 0.0001)	
	B = Placebo		−0.178 (*p* < 0.0001)	
t2 ΔFmax	A = Test		−0.194 (*p* < 0.0001)	
	B = Placebo		−0.175 (*p* < 0.0001)	
t2 ΔQ	A = Test		−0.193 (*p* < 0.0001)	
	B = Placebo		−0.174 (*p* < 0.0001)	
t3 ΔF	A = Test			−0.024 (*p* = 0.529)
	B = Placebo			−0.049 (*p* = 0.1879
t3 ΔFmax	A = Test			−0.025 (*p* = 0.523)
	B = Placebo			−0.048 (*p* = 0.199)
t3 ΔQ	A = Test			−0.024 (*p* = 0.537)
	B = Placebo			−0.048 (*p* = 0.203)

### Results of the visual examination (ICDAS)

At baseline, all included surfaces were detected as caries free (ICDAS 0). Over time, initial lesions (ICDAS 1–2) occurred more frequently in group B, with statistically significant differences at t2 and t3 (Table 7), suggesting a trend toward fewer lesions in group A. Cohen´s effect size at t3 was 0.22 (95 CI 0.14;0.34). Throughout the study, no progression into dentin was observed (that is ICDAS scores 3–6). All results are displayed in Table 7.

**Table 7:** Results of the ICDAS findings in both groups at different investigation times

Investigation time	Group	ICDAS 1/2 n (%)	*p*-value
t1	A = Test	88 (9.1%)	0.076
	B = Placebo	103 (11.9%)	
t2	A = Test	109 (11.4%)	0.016
	B = Placebo	130 (15.0%)	
t3	A = Test	52 (7.2%)	< 0.0001
	B = Placebo	109 (14.5%)	

## Discussion

The present triple-blind, randomized controlled clinical trial assessed the efficacy of a home-applied self-assembling peptide (P11-4 gel with fluoride) in preventing enamel demineralization during multibracket orthodontic treatment. The findings provide clinically relevant evidence supporting the adjunctive use of P11-4 gel in reducing the development and progression of initial carious lesions throughout the course of fixed appliance therapy. This was demonstrated by both visual (ICDAS) and quantitative light-induced fluorescence (QLF) assessments over an 18-month period. Compared to the placebo group, participants using the P11-4 formulation exhibited fewer new lesions, reduced fluorescence loss, and smaller lesion volumes across all investigation times. Visual assessment with ICDAS revealed a significantly lower proportion of tooth surfaces with initial lesions in the test group compared to the placebo group at 12 and 18 months. The results were supported by quantitative measurements with light-induced fluorescence (QLF), which showed significantly better fluorescence values (ΔF, ΔFmax and ΔQ) in the test group throughout the study. Importantly, although both groups exhibited some degree of demineralization, the peptide-treated group showed less lesion development and, in some cases, a reduction in lesion volume (ΔQ), and indicating effective remineralization. These findings are consistent with earlier studies evaluating P11-4 in both office-based and in-situ applications. Jablonski-Momeni et al. [27] demonstrated remineralization effects adjacent to orthodontic brackets using P11-4 under controlled in situ conditions. Welk et al. [29] also reported that P11-4 led to statistically significant reductions in lesion size and fluorescence loss in orthodontically induced initial caries lesions. It is noteworthy that the present study is among the first to confirm the long-term efficacy of the P11-4 gel in a home-based regimen over an extended treatment duration, thereby enhancing its clinical relevance. The observed remineralizing effect of P11-4 may be attributed to its biomimetic mechanism: upon contact with demineralized enamel and a slightly acidic pH, the peptide self-assembles into a 3D matrix that promotes hydroxyapatite nucleation and guided crystal growth [24]. Our data suggest that regular application of the P11-4 gel with fluoride supports sustained enamel remineralization, even in the high-risk context of fixed orthodontic appliances. This is particularly notable given that the placebo gel contained fluoride in an identical concentration to the test gel, with the exception of the peptide. Consequently, the substantial remineralizing effect observed in the test group can be attributed to the presence of P11-4.

Even though fluoride remains the cornerstone of prevention [10, 11], studies suggest that it alone may not be sufficient to prevent initial lesions in orthodontic patients without optimal patient compliance [15] or in high caries risk populations [36]. Biomimetic strategies are beneficial because they function in parts independently of patient behavior and offer complementary mechanisms of action.

It is accepted that the orthodontic environment presents particular challenges for caries prevention due to the increased risk of plaque accumulation and patient-dependent oral hygiene [1, 2]. Some authors even state that high treatment demand and the occurrence of biofilm-related complications make orthodontic treatment a potential public health threat [37]. In the present study, oral hygiene indices of the two groups remained comparable throughout the duration of the study (Fig. 3). The regular use of the peptide was found to compensate for the suboptimal oral hygiene during orthodontic treatment to a significant extent. Furthermore, the current study adds value by demonstrating that P11-4 with fluoride can be effectively delivered in a home-based regimen via a low-concentration fluoride-containing gel. This represents a shift from professionally applied interventions to accessible, patient-managed protocols. The fluoride concentration (900 ppm) is below that of most varnishes, yet the combination with P11-4 appears to be further potentiated by the biomimetic agent P11-4. This is consistent with earlier observations [23, 25–28, 38, 39].

Danisman et al. [17] compared P11-4 with casein phosphopeptide-amorphous calcium phosphate fluoride (CCP-ACPF) in a laboratory-based multi-technique approach and found both effective in preventing lesion progression, but P11-4 showed superior depth remineralization. These results are in line with the present study, where fluorescence loss (ΔF and ΔFmax) and lesion volume (ΔQ) were consistently better in the P11-4 group across all investigation times. A further promising approach is the use of probiotics to reduce mutans streptococci counts in patients undergoing fixed orthodontic treatment. Nevertheless, given the heterogeneity of the studies, it is currently not possible to draw any conclusions about a particular probiotic [18].

In our study, the incidence of demineralization in the population under study was lower than presented in other reports [8]. This is notable given the high caries risk exhibited by the patients, a consequence of both the retentive factors associated with orthodontic appliances and the patients' age. This demonstrates the effectiveness of our prevention approach, including regular dental cleaning and use of fluorides. The management plan for the patient's caries risk factors should be tailored to the individual patient´s needs to involve actions to protect sound tooth surfaces from developing new caries lesions. In order to achieve this, a preventative plan should address home care as well as clinical interventions [32]. Introducing behavior change techniques to orthodontic practice could favorably affect patients’ habits, thus reducing the side effects of treatment [40]. The home-use nature of the P11-4 gel with fluoride may encourage better adherence than more invasive or chairside-only preventive measurements. However, lack of antibacterial activity indicates that it may be used in conjunction with other caries prevention strategies that address bacterial factors.

While most studies on P11-4 have reported favorable outcomes, there are some that present more nuanced or limited findings. Some authors [41] showed that application of self-assembling peptides on demineralized bovine enamel did not lead to increased fluorescence using QLF, indicating either lack of remineralization or irregular crystals. In a randomized controlled trial the remineralizing effect of self-assembling peptide (P11-4), Nano silver Fluoride (NSF) and sodium fluoride (NaF) on initial caries lesions in permanent teeth were compared [42]. It was shown that P11-4 and NSF varnish reduced the ICDAS scores, caries activity, and laser-fluorescence readings of lesions in permanent teeth. However, the change in ICDAS scores was not significantly different from NaF [42]. In a meta-analysis on P11-4 studies a high degree of heterogeneity among studies was highlighted [26]. It was concluded that while P11-4 was generally effective in arresting lesion progression, its superiority over fluoride alone was not always statistically significant in every setting [26]. It should be noted that variations in study outcomes may result from different settings being used, which could compromise the comparability of the results.

Visual examination is generally recommended as the primary method for caries detection on accessible surfaces [43]. In the trial registry, QLF was emphasized as the primary endpoint because it provides an objective and quantifiable measure of early demineralization. Nevertheless, thorough clinical examination is routine at every patient contact, although it was not listed separately in the registration procedure. ICDAS was included from the outset as part of the clinical assessment, since visual caries detection is an integral element of current practice. In this study, ICDAS findings are reported as exploratory secondary outcomes to complement the QLF results and provide additional clinical context. While visual criteria are highly practical in daily care, QLF is more sensitive in detecting initial demineralization before a lesion becomes clinically visible [44]. As visual scoring is inherently operator-dependent, fluorescence-based techniques can serve as a valuable adjunct by providing quantitative data and images, thereby supporting more reliable detection and longitudinal monitoring. This allows a more objective assessment of demineralization compared with visual inspection alone and, in an era of increasing digitization, may also enhance patient communication [45].

One advantage of QLF is that it is non-destructive and allows longitudinal in vivo measurements [46]. The concept of surrogate endpoints has been discussed in the context of fluorescence measurements in dental research when compared to clear indicators of treatment failure or disease. However, waiting for a dentine cavity to appear as true endpoint would neither be practical nor ethical in the context of our study [47]. Nevertheless, QLF continues to be used in research to detect and monitor enamel demineralization [28, 48, 49]. Moreover, no clinical reference standard currently exists that can be considered a non-destructive true gold standard for mineral loss in clinical studies. As with any method, certain limitations must be acknowledged; therefore, our findings should be interpreted with caution. To provide complementary clinical context, QLF outcomes are presented alongside the exploratory ICDAS findings.

One limitation of the present study is that the sample size calculation was not directly based on the anticipated between-group difference in the primary endpoint (ΔF%) but instead relied on clustering assumptions (ICC = 0.05) applied to the trial design. This approach may occasionally be applied in clinical research but does not perfectly align with the continuous outcome measure. To strengthen the interpretation, we therefore calculated and reported effect sizes (Cohen’s d) for all variables. Moreover, we added a post-hoc power analysis based on the observed effect size for ΔF as the primary outcome variable. Despite the methodological limitation, the consistency observed between QLF and ICDAS outcomes supports the robustness of our findings. At t3, the calculated effect sizes indicated small to moderate treatment effects. Cohen´s d values for ∆F and ∆Fmax suggest a moderate effect on lesion fluorescence loss, while the effect size for ∆Q points to a smaller yet consistent effect on lesion area. Taken together, these results indicate that the intervention may contribute to a measurable and potentially clinically relevant reduction in enamel demineralization during orthodontic treatment. However, these findings should be interpreted with caution. The effect sizes, although consistent, remain in the small-to-moderate range, and the confidence intervals, while narrow, do not exclude the possibility of limited clinical impact. Moreover, the modest sample size restricts the generalizability of the results, and further studies with larger cohorts are needed to confirm the robustness and clinical significance of these observations. For the primary outcome ΔF, the post-hoc power analysis yielded an estimated power of 15% at the participant level and 99% at the tooth level. This indicates that, while the study was well powered for the clustered tooth-level analyses (for which the original sample size calculation was performed), the effective power to detect between-group differences at the participant level was low. Patient-level ΔF comparisons must be interpreted with caution due to limited statistical power. In this context, it should be acknowledged that the recruitment of large patient numbers in such trials is not always feasible, and pragmatic constraints play an important role, particularly when multicenter studies are not possible.

A further limitation concerns the duration of follow-up. Although the 18-month observation period was sufficient to detect early enamel changes during fixed orthodontic treatment, it does not allow conclusions about the long-term stability of lesions after the removal of appliances. Longer-term follow-up would be desirable to assess the stability of the effects and to better understand lesion behavior after debonding. However, extended observation is challenging to implement in a pragmatic clinical setting: the study material is associated with costs, patient adherence tends to decline over prolonged periods, and some patients may not remain in fixed-appliance treatment for a longer period. In this context, the present study duration represents a comparatively long and clinically relevant observation window for the selected population.

Another limitation concerns patient adherence, which was assessed through self-reporting. This approach is realistic in clinical research, where objective monitoring of home-care routines over extended periods is not readily feasible. Although oral hygiene indices developed similarly in both groups, indicating comparable home-care performance, the potential for reporting bias cannot be excluded and should be considered when interpreting the results.

While QLF has been accepted to monitor enamel demineralization, its application for assessing plaque accumulation in orthodontic patients was shown to be less reliable. In patients wearing MB appliances, there was no clinically significant agreement regarding the plaque-covered tooth surface depicted by QLF-D respectively conventional images of disclosed plaque [50]. The authors concluded that QLF-D was not reliable for precise plaque quantification in MB patients due to the large method discrepancy [50]. In the present study, the well-known parameters for oral hygiene employed in the Department were used. In future analysis, the available QLF images of the present study can be assessed in order to compare the clinical indices with the fluorescence-based plaque scores.

Gender-specific differences in QLF outcomes were only observed in the placebo group (Group B), where female participants showed significantly lower ΔF and ΔFmax values compared to male participants at t3 (Table 5). The differences were not present in the P11-4 group, suggesting that the active intervention may have reduced interindividual variability, including potential gender-related differences in enamel response or oral hygiene behavior. Moreover, the gender distribution at t3 was unbalanced in group B (7 female, 4 male), which may have contributed to statistical differences due to the small subgroup sizes. The observed gender difference in lesion progression within the placebo group, where female participants exhibited more demineralization, is consistent with previous studies suggesting that hormonal fluctuations and salivary differences may influence caries susceptibility [51]. Gender-specific differences in lesion development would suggest the need for individualized preventive strategies. However, such findings are not easy to interpret in the present study since we did not investigate gender-specific aspects in the patient´s medical histories, nor included this in the initial experimental design. Given the limited sample size, these exploratory findings should be interpreted with caution and warrant further investigation in larger cohorts.

In this study, incisors were the tooth type most affected by demineralization. This is in line with other studies who reported that the highest incidence occurred among the maxillary incisors [52]. Others reported in contrast that caries prevalence was lower in incisors and cuspids than in molars and premolars [53]. Nevertheless, a thorough examination of all teeth, tooth surfaces, and gingiva is crucial during orthodontic treatment, irrespective of the tooth type and surface. Consequently, the implementation of preventative measures can be carried out in a sufficient timely manner.

It is also worth noting that in recent times the link between preventive dentistry and sustainability is gaining attention [54]. By reducing the need for resource-intensive treatments and minimizing waste, prevention helps preserve natural tooth structures and supports long-term oral health, contributing to a more sustainable healthcare system.

Despite the strength of the randomized, triple-blind design, a few limitations must be considered. Patients’ compliance was self-reported, though supported by regular reviews and consistency in hygiene indices across both groups. Oral hygiene adherence, a known confounder in clinical caries research, was monitored through standardized indices and showed no significant intergroup differences, reducing this concern. A further limitation is the 18-months study duration, which, while adequate for the detection of early caries changes, does not allow conclusions regarding long-term lesion stability post-treatment. Longer-term follow-up is essential to assess the permanence of remineralization and to observe lesion behavior post-orthodontic treatment.

## Conclusion

So far, only limited evidence was existing regarding the effectiveness of P11-4 gel with fluoride as a home-based preventive measure during the high-risk period of fixed orthodontic treatment. The present study contributes relevant data to this gap and, taking into account the limitations discussed above, leads to the following conclusions:

- Regular home use of P11-4 gel with fluoride reduced the development and progression of initial enamel lesions during multibracket orthodontic treatment compared to placebo.
- The accuracy of the data was confirmed through both visual examination and fluorescence-based measurements.
- Despite the small sample size and limited patient-level power, integration of P11-4 with fluoride into home-care regimens represents a promising adjunct to fluoride-based caries prevention, especially during orthodontic treatment with fixed appliances.
- Larger trials are required to confirm the clinical relevance of these effects.

## Data Availability

All data generated or analyzed during this study are included in this article. The dataset will be available in an institutional data repository after publication. The repository link can be obtained from the corresponding author upon reasonable request.

## Abbreviations

API: Approximal Plaque Index
CCP-ACPF: Casein Phosphopeptide-Amorphous Calcium Phosphate Fluoride
CONSORT: Consolidated Standards of Reporting Trials
DRKS: German Clinical Trials Register
GDPR: General Data Protection Regulation
ICC: Intraclass Correlation Coefficient
ICDAS: International Caries Detection and Assessment System
MB: Multibracket
NaF: Sodium Fluoride
NSF: Nano Silver Fluoride
QLF: Quantitative Light-induced Fluorescence
SBI: Sulcus Bleeding Index
